# Autonomic cardiovascular dysregulation as a potential mechanism underlying depression and coronary artery bypass grafting surgery outcomes

**DOI:** 10.1186/1749-8090-5-36

**Published:** 2010-05-13

**Authors:** Tam K Dao, Nagy A Youssef, Raja R Gopaldas, Danny Chu, Faisal Bakaeen, Emily Wear, Deleene Menefee

**Affiliations:** 1 University of Houston, 4800 Calhoun Rd., Houston, TX 77004, USA; 2 Michael E. DeBakey Veterans Affairs Medical Center, Texas Medical Center, 2450 Holcombe Blvd., Suite 1, Houston, TX 77021, USA; 3 Department of Psychiatry and Behavioral Sciences, Baylor College of Medicine, One Baylor Plaza BCM 350, Houston, TX 77030, USA; 4 Department of Surgery, Division of Cardiothoracic Surgery, Baylor College of Medicine, 1709 Dryden, Suite 1500, Houston, TX 77030, USA; 5 Department of Psychiatry and Behavioral Sciences, Duke University Medical Center, Durham, NC 27710, USA; 6 VA Mid-Atlantic Mental Illness Research, Education, and Clinical Center, Durham; 7 VA Medical Center, Durham, NC 27705, USA; 8 University of South Alabama, College of Medicine, Mobile, Alabama 36617, USA

## Abstract

**Background:**

Coronary artery bypass grafting (CABG) is often used to treat patients with significant coronary heart disease (CHD). To date, multiple longitudinal and cross-sectional studies have examined the association between depression and CABG outcomes. Although this relationship is well established, the mechanism underlying this relationship remains unclear. The purpose of this study was twofold. First, we compared three markers of autonomic nervous system (ANS) function in four groups of patients: 1) Patients with coronary heart disease and depression (CHD/Dep), 2) Patients without CHD but with depression (NonCHD/Dep), 3) Patients with CHD but without depression (CHD/NonDep), and 4) Patients without CHD and depression (NonCHD/NonDep). Second, we investigated the impact of depression and autonomic nervous system activity on CABG outcomes.

**Methods:**

Patients were screened to determine whether they met some of the study's inclusion or exclusion criteria. ANS function (i.e., heart rate, heart rate variability, and plasma norepinephrine levels) were measured. Chi-square and one-way analysis of variance were performed to evaluate group differences across demographic, medical variables, and indicators of ANS function. Logistic regression and multiple regression analyses were used to assess impact of depression and autonomic nervous system activity on CABG outcomes.

**Results:**

The results of the study provide some support to suggest that depressed patients with CHD have greater ANS dysregulation compared to those with only CHD or depression. Furthermore, independent predictors of in-hospital length of stay and non-routine discharge included having a diagnosis of depression and CHD, elevated heart rate, and low heart rate variability.

**Conclusions:**

The current study presents evidence to support the hypothesis that ANS dysregulation might be one of the underlying mechanisms that links depression to cardiovascular CABG surgery outcomes. Thus, future studies should focus on developing and testing interventions that targets modifying ANS dysregulation, which may lead to improved patient outcomes.

## Background

It is estimated that 16 million American adults have coronary heart disease (CHD). CHD remains the leading cause of death in the United States with 652,091 registered deaths in 2005 [1]. To date, multiple longitudinal and cross-sectional studies have examined the association of CHD with psychological functioning, particularly depression [2,3]. Over 100 studies have investigated this relationship, thus providing evidence that depression is prevalent (18% to 60%) in patients with CHD. This comorbidity has significant adverse effects on the course and outcome of CHD [4-7]. Depressed patients are twice as likely as nondepressed patients to have a major cardiac event within 12 months of the diagnosis of coronary artery disease [8]. In addition, the risk of mortality is greater in depressed patients compared to nondepressed after the following events: CHD [4], acute myocardial infarction [9], an episode of unstable angina [10], or CABG [4,5].

Although the relationship between depression and cardiac events is well established, the mechanism underlying this relationship remains unclear [11]. However, three lines of evidence suggest that altered autonomic nervous system (ANS) activity in depressed patients might be responsible for the increased risk of mortality and medical morbidities in patients with CHD. First line of evidence originates from early reports of ANS dysregulation in depression was found in studies of medically ill patients with major depressive disorder (MDD). These studies found elevated levels of plasma and urinary catecholamines, primarily norepinephrine (NE), in depressed patients compared with controls [12-14]. These findings are significant because the concentrations of plasma NE generally parallel the level of activity of the sympathetic nervous system (SNS) and are highly correlated with sympathetic neural activity [14].

A second line of evidence is based on the consistent findings that resting Heart Rate (HR) is higher in depressed than nondepressed patients [14-16]. Depression is also associated with exaggerated HR response to physical and psychological stressors in both medically well individuals [17] as well as in patients with CHD [18]. As regulation of HR occurs primarily through a reciprocal interaction of the sympathetic and parasympathetic nervous system, and given that one of the functions of ANS is to regulate HR, elevated HR suggests dysregulation of cardiac ANS function.

A third line of evidence is based on studies reporting decreased Heart Rate Variability (HRV) among depressed patients compared to nondepressed controls [8,19,20]. Over the last two decades, HRV has emerged as an important marker for examining the continuous interplay between the parasympathetic and sympathetic influences on HR that yields information about autonomic flexibility [21]. Increased HRV has been used as a marker of increased vagal activity and has been consistently associated with greater capacities to regulate stress, emotional arousal, and attention [22] while low HRV has been associated with excessive cardiac sympathetic modulation, inadequate parasympathetic modulation, or both [23]. A number of studies have found HRV to be lower in depressed psychiatric patients compared to controls [20,21]. There is even greater evidence that HRV is lower in depressed than nondepressed patients with CHD [24,25].

In summary, there is considerable evidence of autonomic cardiovascular dysregulation in depressed patients as well as in patients with CHD. However, it is unknown whether patients with CHD and depression have greater ANS dysregulation relative to patients with either depression or CHD alone (i.e., comorbidity versus single morbidity). It is also unknown whether ANS dysregulation explains the increased morbidity and mortality in patients with both disorders. Thus, the purpose of this study was twofold. First, we compared three markers of ANS function in four groups of patients: 1) Patients with coronary heart disease and depression (CHD/DEP), 2) Patients without CHD but with depression (NonCHD/Dep), 3) Patients with CHD but without depression (CHD/NonDep), and 4) Patients without CHD and depression (NonCHD/NonDep). Second, we investigated the association of ANS activity (HR, HRV, and plasma NE levels) impact of depression and autonomic nervous system activity on CABG outcomes.

Second, we investigated the association between these markers of ANS function and group classification in cardiac patients (i.e., CHD/DEP vs. CHD/NonDep) and CABG outcomes (i.e., in-hospital length of stay and patient's type of discharge (i.e., routine or nonroutine), while holding constant potential differences in medical (e.g., diabetes, history of myocardial infarction, etc.) and sociodemographic (e.g., age, gender, etc.) variables. We hypothesized that patients in the CHD/Dep group will have the greatest dysregulation in autonomic function while patients in the NonCHD/NonDep group will have the least amount of autonomic dysregulation compared to the other 2 groups. We also hypothesized that ANS markers and group classification in cardiac patients will significantly predict in-hospital length of stay and patient's type of discharge. Specifically, there will be a significant positive association between HR and plasma NE levels and in-hospital length of stay. There will be a significant negative association between HRV and in-hospital length stay. In addition, patients in the CHD/Dep group will more likely be discharged non-routinely discharged following a CABG operation than those with CHD only. Both of these hypotheses reflect a possible additive effect of depression and heart disease on ANS dysregulation.

## Methods

### Participants

A sample of patients was recruited from private sector hospitals in the Northeast to form four groups of patients: 1) Patients with CHD and depression (CHD/DEP), 2) Patients without CHD and with depression (NonCHD/Dep), 3) Patients with CHD and without depression (CHD/NonDep), and 4) Patients without CHD and depression (NonCHD/NonDep). It should be noted that patients without depression have no current major depressive episodes. Patients with a history of depression, or minor forms of depression may be included in the nondepressed group.

### Procedure

Patients in the CHD/Dep and CHD/NonDep groups were recruited from patients who have a CHD diagnosis and were scheduled to undergo a first-time CABG with or without concomitant valve procedure. Patients in the NonCHD/NonDep group were recruited from a primary care clinic within the hospital while patients in the NonCHD/Dep group were recruited from the hospital's outpatient mental health clinics. Those who consented to participate in the study were assessed to determine if they met the study eligibility criteria. The inclusion criteria for the CHD/Dep group consisted of being enrolled to undergo a CABG operation, and having a diagnosis of MDD. The exclusion criteria for the CHD/DEP group were significant cognitive deficits or other psychiatric diagnoses. The inclusion criterion for the CHD/NonDep group consisted of being enrolled to undergo a CABG operation. The exclusion criteria for the CHD/NonDep group consisted of significant cognitive deficits, a diagnosis of MDD, or any other psychiatric diagnosis. The inclusion criterion for the NonCHD/Dep group consisted of a diagnosis of MDD. The exclusion criteria for this group consisted of a diagnosis CHD, significant cognitive deficits, or any other psychiatric diagnosis. Patients in the NonCHD/NonDep group were excluded if they had a diagnosis of MDD, CHD, significant cognitive deficits, or any other psychiatric diagnosis.

### Screening

Patients were initially screened to determine whether they met inclusion or exclusion criteria. Psychiatric interview and a psychophysiological assessment were conducted on all subjects who consented to participate in the study.

### Psychiatric Interview

The MINI International Neuropsychiatric Interview (MINI) [26] is a standardized diagnostic instrument for the diagnosis of psychiatric disorders using the Diagnostic and Statistical Manual, 4^th ^edition (DSM-IV-TR) [27] and International Classification of Diseases (ICD) - 10 psychiatric disorders [28]. It consists of standardized, structured, closed-end questions throughout its diagnostic procedure The MINI has demonstrated adequate reliability and validity. Inter-rater and test-retest reliabilities were high among the majority of disorders. Validities with other lengthy structured diagnostic interviews such as the Structured Clinical Interview (SCID) for DSM-IIIR have been reported [26]. Research has shown that the MINI can be used successfully as a gold standard of psychiatric diagnosis in multi-center clinical trials and epidemiology studies [29]. The MINI was used to make the diagnosis of MDD.

### Heart Rate and Heart Rate Variability Measurement

After a 12 hour fast which includes abstinence from smoking and a seated rest of 30 minutes, the HR, HRV and plasma NE levels were measured for each subject. The assessment of HR and HRV were gathered via recordings of EKG and respiration using the Nexus 10 BioTrace equipment and associated software version 1.16. The Nexus 10 is a 10 channel physiological monitoring and feedback platform that offers data acquisition at up to 2048 samples per second. It is a certified class 2-1 (EU) medical device. Following previous conventions [30], patients were excluded from further analysis if they were not in predominantly regular sinus rhythm or if they had sustained atrial arrhythmias such as atrial fibrillation or greater than 10% ectopic complexes. During EKG measurement, participants were instructed to maintain open eyes and avoid moving their wrists while the experimenter read excerpts from a collection of pleasant travel stories. This is a common HRV experimental paradigm designed to mimic normal waking state levels of arousal [31]. HRV was recorded for 15 minutes for each participant. At the end of the session the recordings were coded and saved for subsequent analysis. Movement artifacts above a certain threshold were automatically removed from the session overview which provides a display of the total session of respiration and heart rate data. Following previous convention [32], heart rate data were averaged across 60 seconds intervals at a sampling rate of 512 hertz and edited by averaging premature ectopic beats that exceeded a 25% difference between two consecutive data points. HRV was calculated as the standard deviation of all normal-to-normal RR intervals (SDNN; intervals between adjacent QRS complexes).

### Plasma Norepinephrine Assessment

Blood samples (1.2 mL) were drawn from the antecubital vein by acute venipuncture and were contained in chilled, heparinized tubes containing ethylene glycol tetraacetic acid and 200 mmol/L reduced gluthione. The plasma was then stored in polystyrene tubes at −70°C until assayed. The assay and laboratory procedures for measuring NE have been described in detail elsewhere [33], and have been used by other investigators in similar studies [34].

#### Medical Covariates

A number of plausible variables has been identified that could influence ANS regulation, particularly HR, HRV, and plasma NE levels. To help partition out the effects of these variables, we have included the following covariates: age, education, race, diabetes mellitus, hypertension, history of asthma, history of myocardial infarction, cigarette smoking, alcohol consumption, level of physical activity, body mass index (BMI), and the Deyo score [35].

The Deyo Score is a comorbidity index that was adapted from the Charlson Comorbidity Index [36]. It is designed to capture comorbid conditions recorded in the inpatient setting using ICD-9-CM diagnosis and procedure codes and has been widely used in outcomes studies with administrative datasets as the principal data source[37]. The Deyo Score assesses comorbid medical conditions such as myocardial infarction, congestive heart failure, peripheral vascular disease, cerebrovascular disease, dementia, chronic obstructive pulmonary disease, rheumatologic disease, mild liver disease, diabetes melllitus, diabetic complications, hemiplegia or paraplegia, renal disease, malignancy, moderate to severe liver disease, metastatic solid tumors, and acquired immune deficiency syndrome/human immunodeficiency viral infection. The Deyo Score was determined by weighted scoring of comorbidities. Individual comorbidities were then combined to form a Deyo Score [35].

Patients were considered smokers if they smoked1 pack or more of cigarettes per week and for at least 5 years were considered smokers. BMI was assessed using a BMI calculator that took into consideration the patient's weight and height. Physical activity was assessed using the International Physical Activity Questionnaires (IPAQ) [38]. The IPAQ consists of eight items that estimate the time spent performing physical activities (low to high). A number of studies have been conducted on the IPAQ, showing that it produces reliable data as well as acceptable concurrent, criterion, and construct validity [38,39].

Alcohol consumption was measured using the Alcohol Use Disorders Identification Test (AUDIT) [40]. The AUDIT is a 10-item self-report questionnaire. Each of the questions has a set of responses to choose from, and each response has a score ranging from 0-4. All the responses were then added to form a total score. A recent systematic review of the literature has concluded that the AUDIT is the best screening instrument for the range of alcohol problems in primary care [41].

### Outcome Variables

Outcome variables in this study included length of inpatient hospital stay and patient disposition. Length of inpatient hospital stay (measured in days) is defined as the difference between the hospital admission date and the date of discharge for the patient. Disposition of patients was coded as routine or non-routine. Patients were coded as having a non-routine disposition if they were discharged to short-term hospital, skilled nursing facility, intermediate care facility, another type of facility, home health care, or against medical advice.

### Statistical Analysis

We hypothesized that patients in the CHD/Dep group will have the greatest dysregulation in autonomic function while patients in the NonCHD/NonDep group will have the least amount of autonomic dysregulation compared to the other 2 groups. To examine our first hypothesis, chi-square and one-way analysis of variance (ANOVA) were performed to evaluate group differences across demographic and medical variables, as well as markers of ANS dsyregulation. Any variables that differed significantly between the four groups were used in subsequent regression models as covariates to assess the independent impact of ANS indicators on medical outcomes following CABG. Our second hypothesis was that ANS markers and group classification of cardiac patients (see above) will significantly predict in-hospital length of stay and patient discharge disposition. Specifically, there will be a significant positive association between HR and plasma NE levels and in-hospital length of stay. There will be a significant negative association between HRV and in-hospital length stay. In addition, patients that are in the CHD/Dep group will more likely be discharged non-routinely following a CABG operation than those in the CHD/NonDep group. To address these hypotheses, logistic regression and multiple regression analyses were used. Logistic regression analysis was conducted with patient discharge disposition as an outcome variable after controlling for the effects of age, Deyo score, physical activity, and BMI (i.e., variables that were significant in previous chi-square and ANOVA analyses). Independent variables in this analysis include group membership, HR and HRV. Multivariable regression analysis was also performed assessing the impact of group membership, HR, and HRV on in-hospital length of stay after controlling for the effects of age, Deyo score, physical activity, and BMI.

## Results

Chi square test and separate one-way ANOVAs were conducted to evaluate the relationship between groups of patients and demographic and medical characteristics (See Table 1). The independent variable had four levels: CHD/Dep, CHD/NonDep, NonCHD/Dep, andNonCHD/NonDep. The dependent variables were demographic, medical, and ANS dysregulation variables. For age, the ANOVA was significant, *F*(3, 358) = 3.75, *p *= .011. The strength of the relationship between groups of patients and age, as assessed by η^2^, was weak, with the groups of patient factor accounting for 1% of the variance of the dependent variable. For the Deyo score, the ANOVA was significant, *F*(3, 358) = 5.59, *p *= .001. The strength of the relationship between groups of patients and the Deyo score was weak with the groups of patient factor accounting for 4.5% of the variance of the dependent variable. For BMI, the ANOVA was significant, *F*(3, 358) = 7.46, *p *< .001. The strength of the relationship between groups of patients and the BMI was weak with the groups of patient factor accounting for 5.9% of the variance of the dependent variable. The four groups also differ on physical activity, *χ^2 ^*(3, *n *= 362) = 45.6, *p *< .05 (two-tailed), with *ϕ *= .067. For heart rate, the ANOVA was significant, *F*(3, 358) = 13.3, *p *< .001. The strength of the relationship between groups of patients and HR was weak, with the groups of patient factor accounting for 16% of the variance of the dependent variable. For HRV, the ANOVA was significant, *F*(3, 358) = 205.1, *p *< .001. The strength of the relationship between groups of patients and HR was strong, with the groups of patient factor accounting for 46% of the variance of the dependent variable.

**Table 1:** Demographic and Medical Characteristics

Characteristics	CHD/Dep (1)	NonCHD/Dep (2)	CHD/NonDep (3)	NonCHD/NonDep (4)	*p*	Post Hoc
Age	61.3 (8.3)	59.2 (9.1)	62.0 (9.3)	58.3 (7.5)	.011	3 > 4*
Education	10.3 (4.9)	12.1 (6.3)	12.2 (7.1)	11.9 (6.6)	.169	
Race					.326	
Caucasian	81.9% (68)	79.3% (73)	81.4% (79)	91.2% (82)		
African American	9.6% (8)	14.2% (13)	10.3% (10)	6.6% (6)		
Hispanic	8.5% (7)	6.5% (6)	8.3% (8)	2.2% (2)		
Deyo score	1.39 (1.04)	.961 (1.35)	1.30 (.933)	.776 (1.23)	.001	1 > 4* 3 > 4*
History of MI	34.9% (29)	35.9% (33)	35.1% (34)	20% (18)	.062	
History of asthma	3.6% (3)	5.4% (5)	7.2% (7)	4.4% (4)	.724	
Cigarette Smoker	55.4% (46)	59.7% (55)	57.3% (56)	42.2% (38)	.054	
AUDIT	25.8 (6.2)	27.4 (7.2)	26.4 (5.8)	19.6 (4.3)		
Physical Activity					.003	
Low	63.9% (53)	62.0% (57)	51.5% (50)	35.6% (32)		
Moderate	26.5% (22)	33.7% (31)	37.1% (36)	50.0% (45)		
High	9.6% (8)	4.3% (4)	11.3% (11)	14.4% (13)		
Diabetes	26.5% (22)	25% (23)	28.9% (28)	13.3% (12)	.064	
Hypertension	32.5% (27)	28.2% (26)	30.9% (30)	16.7% (15)	.073	
Body mass index (BMI)	29.7 (7.2)	29.3 (6.4)	27.8 (8.8)	24.9 (8.2)	< .001	1 > 4* 2 > 4*
Heart rate	76.3 (11.4)	74.4 (12.2)	71.4 (10.9)	66.9 (10.3)	< .001	1 > 3 > 4* 2 > 4*
Heart rate variability^a^	19.79 (7.9)	24.53 (7.6)	24.89 (7.88)	50.51 (12.5)	< .001	1 < 2 < 4* 1 < 3 < 4*
Plasma NE^b^	293 (99)	343 (175)	308 (211)	341 (160)	.120	

Note. AUDIT = Alcohol Use Disorders Identification Test. CHD/Dep = Patients with CHD and depression. NonCHD/Dep = Patients without CHD but with depression. CHD/NonDep = Patients with CHD but without depression. NonCHD/NonDep = Patients without CHD or depression. ^a^Standard deviation of RR (msec). ^b^alog transformed pg/ml. * *p *< .05.

Follow-up tests were conducted to evaluate the pairwise differences among the means. Because the variances among the four groups ranged from 55.5 to 85.7, we chose not to assume the variances were homogeneous and conducted post hoc comparisons with the use of the Dunnett's *C *test, a test that does not assume equal variances among the four groups. For age, there was a significant difference in the means between the CHD/NonDep and the NonCHD/NonDep groups with the CHD/NonDep group being older than the NonCHD/NonDep group. For the Deyo score, there were significant differences in the means between the CHD/Dep and NonCHD/NonDep groups and the CHD/NonDep and the NonCHD/NonDep groups with the CHD/DEP and the CHD/NonDep groups having higher means scores on the Deyo score compared to the NonCHD/NonDep group. For the BMI, there were significant differences in the means between the CHD/Dep+Dep+CHD and NonCHD/NonDep groups and between the NonCHD/Dep and the NonCHD/NonDep groups with the CHD/DEP and the NonCHD/Dep groups having higher means scores on the BMI compared to the NonCHD/NonDep group. For heart rate, the CHD/DEP group had the highest HR followed by the CHD/NonDep and the NonCHD/NonDep groups. For HRV, the CHD/Dep group had the lowest HRV while the NonCHD/NonDep group had the highest HRV.

Table 2 contains results of logistic regression analysis with patient discharge disposition (non-routine = 1 and routine = 0) as an outcome variable after controlling for the effects of age, Deyo score, physical activity, and BMI. Independent significant predictors of patient discharge were the following: being in the CHD/Dep group (OR: 1.43, HR (OR: 1.39), and HRV (OR: .597). Table 3 contains results of multivariable regression analysis with length of in-hospital stay as the dependent variable. The adjusted *R*^2 ^of .26 indicates that a fourth of the variability in length of stay is predicted by group, HR, and HR variability. Independent significant predictors included: group classification (*B *= 1.56), HR (*B *= .058), and HRV (*B *= .-963).

**Table 2:** Logistic Regression Analysis Predicting Routine Discharge after controlling for the Effects of Age, Deyo score, Physical Activity, and BMI (n = 180)

Variable	*B*	SE *B*	Wald's Statistic	Odds Ratio (95% CI)
Group (1 = CHD/DEP, 0 = CHD/NonDep)	.516*	.023	14.3	1.43 (1.33-2.63)
Heart rate	.343*	.121	15.5	1.12 (1.02-1.04)
Heart rate variability	-.513**	.094	19.9	.597 (.497-.718)

Note. CHD/DEP = coronary artery disease (CHD) and depression. - CHD/NonDep = CHD but without depression. * *p *< .05. ** *p *< .01.

**Table 3:** Multiple Regression Analysis Predicting In-Hospital Length of Stay after controlling for the Effects of Age, Deyo score, Physical Activity, and BMI (n = 180)

Variable	*B*	SE *B*	β	95% CI
Group (1 = CHD/DEP, 0 = CHD/NonDep)	1.56**	.276	.786	.986-2.76
Heart rate	.058*	.096	.265	.456-.956
Heart rate variability	-.963*	.123	-.564	-.126-.021

Note. CHD/DEP = coronary artery disease (CHD) and depression . - CHD/NonDep = CHD but without depression. * *p *< .05. ** *p *< .01.

## Discussion

Despite the significant contribution in the literature on mental health and cardiovascular diseases, we simply do not know at this time which mechanisms account for the relationship between depression and outcomes following a CABG surgery [11]. Also, to the best of our knowledge, there are no published studies that compared the incidence of ANS dysregulation in patients with both CHD and depression, to those with either depression or CHD alone. It is also unknown whether ANS dysregulation could explain CABG outcomes. These two questions are important to address because if ANS dysregulation is what links depression CABG outcomes, then recognition and treatment of ANS dysregulation may lead to improved patient outcomes. Thus, it was in this framework that we sought to address ANS dysregulation and outcomes following a CABG operation.

Our initial analyses revealed that age, Deyo score, physical activity, BMI, HR, and HRV were significantly different across the four groups. Specifically, patients that had CHD only were significantly older than the patients who did not have CHD or depression. Also, those that had CHD with or without depression have higher Deyo scores than patients who did not have CHD or depression. This is expected given that the Deyo score reflects 17 comorbid medical conditions.

The measurement of ANS regulation/dysregulation has long been debated in the medical community. In our study, we defined ANS dysregulation as having a high basal HR, low HRV, and high plasma NE levels. Based on this definition, we found that patients with both depression and heart disease have the greatest autonomic dysregulation compared to the other three groups. The results supported our first hypothesis showing that patients diagnosed with both CHD and depression have HR and lower HRV than patients in the other three groups. However, the findings were not consistent for plasma NE levels; this unexpected finding might be due to the following reasons: It is well documented that there are many factors that can influence plasma levels of catecholamines such as psychological stress, temperature, posture, exercise, medications, and food intake [42]. Furthermore, the sympathetic nervous system consists of many different nerves that are distributed throughout the body. Consequently, the measurement of sympathetic nerve activity in one area of the body may not truly reflect sympathetic nerve activity throughout the body. Moreover, we collected blood samples from the antecubital vein. Given that sympathetic nerve activity in the arm may influence levelsof antecubital plasma NE levels, this measurement of plasma NE level may not accurately reflect plasma NE levels throughout the body. As suggested by others [43,44], total body sympathetic nerve activity might be better assessed using arterialized venous sampling and plasma NE kinetic techniques that rely on dilution of radiolabeled NE and mathematical modeling to provide estimates of postganglionic norepinephrine release and clearance.

The results supported our initial hypothesis that there are group differences across indicators of ANS activity. However, group differences do not provide much support that ANS dsyregulation predicts outcome following a CABG operation. Thus, in our subsequent research questions we examined whether markers of ANS activity predicted in-hospital length of stay and patient discharge disposition. It was found that there was increased length of stay and greater likelihood of having a non-routine discharge following CABG in patients with both depression and CHD compared to those with CHD only. This finding is interesting for the following reasons. First, it suggests that HR and HRV may have an additive effect to CABG outcomes. Second, by including group classification as an independent variable in our analyses (and after controlling for potential confounding variables), we were able to assess whether there was an association between group classification and outcomes following a CABG operation.

Despite the positive findings, there are several limitations to consider, the first of which involves extraneous variables that might inflate the systematic error in the study. Despite statistically controlling for a number of extraneous variables to remove some of the variability in the dependent variable that is due to these extraneous variables, there were other variables that were not controlled for during the study. Because of the nature of the study, which limits the ability to randomly assign patients to different conditions, variables such as the number of grafts patients received and medications that they were currently taking might have a systematic effect on (correlates with) length of stay and patient discharge disposition. Secondly, although this study used the MINI in diagnosing patients, it did not assess reliability of diagnoses using multiple raters. Thirdly, the generalizability of the results of this study to the general population is limited because the study had only male patients and it included patients with depression but without comorbid psychiatric disorders. And Finnaly, the index that was used in this study was SDNN. SDNN is an acceptable measure of HRV for short term measurements. There have been studies of short term HRV as predictors of cardiac mortality and morbidity. However, most studies of HRV and depression in CHD has calculated HRV from 24 hour ambulatory monitoring and used frequency domain indices of HRV.

## Conclusions

In summary, the current study presents evidence to support the hypothesis that ANS dysregulation might be one of the underlying mechanisms that links depression to CABG outcomes. However, further research is needed to control for other potential covariates such as diet and testing conditions to confirm that ANS dysregulation is the mechanism underlying these two conditions. Also, these preliminary results suggest that we begin to focus on treatment-related questions. For instance, future studies should focus on developing and testing interventions that targets modifying ANS dysregulation. Furthermore, it would be beneficial to know if improved ANS regulation can decrease morbidity and mortality in depressed CHD patients following CABG. This line of research may guide therapeutics especially that HRV can be modified through pharmacologic and biobehavioral therapies as well as exercise and exercise therapies [45].

## Competing interests

The authors declare that they have no competing interests.

## Authors' contributions

TD was involved in developing the intellectual content of the manuscript as well as participated in the collection of the data, the analysis of the data, and the drafting of the manuscript. JS was involved in the design of the study as well as participated in the data analysis. EW was involved in revising the important intellectual content of the manuscript. DM participated in the design of the study and drafting the manuscript. EW participated in collecting the data and scoring the instruments. All authors read and approved the final manuscript.
